# Social work, mental health, older people and COVID-19

**DOI:** 10.1017/S1041610220000873

**Published:** 2020-05-12

**Authors:** John Brennan, Patrice Reilly, Kerry Cuskelly, Sarah Donnelly

**Affiliations:** 1 International Federation of Social Workers, Rheinfelden, Switzerland; 2 HSE CHO 9, Dublin, Ireland; 3 HSE Adult Mental Health Services, Dublin, Ireland; 4 University College Dublin, Dublin, Ireland

## Social work, mental health, and older people

In this commentary, we will explore the work of social work in Ireland in addressing the impact of the coronavirus 2019 (COVID-19) crisis on older people in general, and older people who have an enduring mental illness.

## COVID-19: the Irish context

The Republic of Ireland has a population of just under 5 million people. The percentage of people over age 65 years is approximately 20% (CSO, 2016). Ireland has directed that all citizens over 70 years of age should “cocoon” and should not leave their homes and should minimize all non-essential contact with other members of their household (Department of Health, 2020a). Similar to many other countries, Ireland has experienced the now familiar effects of COVID-19, in that (i) it affects mostly older people and those with underlying medical conditions, (ii) there are significant clusters of the virus in nursing homes, (iii) there is a lack of testing facilities, and (iv) there has been a veritable scramble to get adequate supplies of personal protective equipment (PPE). Nursing homes have had great difficulty securing such PPEs and other necessary equipment. Maintaining staffing levels are a continuing cause of concern.

On the positive side, a significant effort has been made by government with the support of all politicians, health and social care authorities, frontline workers and the general public to combat the spread of the virus. A “lockdown” was initiated in mid-March 2020 and at the time of writing, this has been adhered to by the public. Thankfully, the health service has managed to cope with the numbers of people requiring critical medical care.

## Social work with older people in Ireland: the pre-COVID-19 context of practice

Legislation and service provision for older people in Ireland have been given low priority until recent years which, it could be reasoned, is reflective of the systemic and legislative discrimination (Donnelly and O’Loughlin, 2015). Recently, however, there are indications that aging is beginning to be constructed in a more positive framework and older people are now almost universally perceived as a deserving group who are entitled to better quality and increased resourcing of services and support from the State (Scharf *et al*., 2013).

In Ireland, social workers have a key role in the care of older people. They work in general health and mental health and social care settings within the hospital, community, and residential and palliative care sectors. The largest number of such social workers are based in the acute general hospital setting. Significantly, social workers in Ireland and across Europe regularly work with people, families, and/or groups who are in crisis. They work in the gray areas of confusion, uncertainty, and doubt (Jordan, 2000) – nouns that can aptly fit the COVID-19 context very well.

Within the context of COVID-19 in Ireland, it should be noted that while social workers are employed in public residential care units, they are rarely employed in the nursing homes which are primarily run by the private sector. Social workers engaging with the general population of older people work with people presenting with a range of issues including mental health needs. Increasingly, social workers are likely to work with those older people who experience poverty, ill-health, depression, dementia, substance abuse, or those with unresolved traumas from previous years (Phillips and Ray, 2012). Issues of loss and grief are regularly to the fore in such situations. Working with the whole system including families and informal carers is also a key aspect of the work. There are also a subset of social workers working in specific psychiatric settings.

## Social work in mental health services/psychiatry

The types of mental health difficulties manifest in older people in Ireland are similar to that found elsewhere in the world. Older people with mental health difficulties can present with anxiety and depression, dementia-related behaviors, and problems arising from alcohol misuse; a smaller cohort of older people can present with late-onset psychosis or schizophrenic type conditions. The social workers in the specialist mental health subset are based in community mental health teams and in acute psychiatric hospital and residential care settings. A further subset comprises social workers working on psychiatry of later life teams.

The precise mental health social work role on the older persons’ mental health teams is significantly a family-focused role. A large part of the support/intervention is in relation to family support, especially concerning persons with cognitive impairment, such as dementia. Interventions will vary depending on the underlying mental health difficulty.

## What has changed?

The COVID-19 pandemic has exacerbated many issues for older people and their carers, while throwing up new difficulties, particularly an increase in mental health difficulties (Galwa *et al*., 2020). Ageism, human rights, social justice, and ethical dilemmas form a backdrop to, for example, experiences of illness, death and dying, grief and loss, isolation, and safety concerns. Additionally, there is a reduction in formal support from service providers during these unprecedented times. For older people generally, COVID-19 has hugely challenged their opportunities to exercise self-determination in realizing their own well-being. For example, older people who are vulnerable because of a mental health problem may be further excluded from decision-making about their own care needs or plans. Given the underlying levels of ageism in society in Ireland and elsewhere, social justice was a key issue in pre COVID-19 times at both the individual and policy levels. However, such concerns have been exacerbated even further by the present extraordinary circumstances resulting from COVID-19.

### For older people in nursing/residential homes

For older people in nursing homes, they are at an increased risk of contracting the COVID-19 virus. At the time of writing, the virus is present in one-third of nursing homes in Ireland with 50% of all COVID-19-related deaths occurring in these settings (Department of Health, 2020b). When care homes have residents who have dementia, and who may be prone to wandering, risks of spreading the illness increase even more. A further concern is that such residents will be given antipsychotics to sedate them which in itself is a risk; such practices are also abusive and a denial of rights and a deprivation of liberty. Risk of abuse is even greater when there are replacement or temporary staff and given the absence of visiting families who can act as an aid to monitoring their loved one’s health and well-being. In addition, isolation has increased for all residents because visiting has largely stopped leading to increased loneliness as a result with a concomitant increase in depressive feelings and anxiety levels.

The overstretched care staff themselves are at risk of severe burnout, precipitating perhaps a further threat of abusive care practices. This risk is further increased because many Safeguarding and Protection Social Workers in Ireland have been redeployed to “Contact and Tracing” teams meaning that there are significantly fewer frontline social workers to investigate and monitor abusive situations. Also, many homes are being managed by agency staff due to staff sick leave. Such sick leave absences have left some nursing homes with difficulties getting replacement staff, thereby impacting on the general care of the residents. Aside from the physical care that is potentially diminished, the human relationships formed between residents and regular staff are consequently broken in these circumstances.

COVID-19 restrictions also impact on the rights of residents including end-of-life wishes which may not be sought or be disregarded entirely. Funeral rituals, traditions, and arrangements have been for the most part entirely disrupted. Residents are often dying without the presence of close family members. Family members therefore do not have the opportunity to say good-bye before losing their loved one unless a phone or video call can be facilitated. After death, coffins are now closed when COVID-19 has been diagnosed so family members do not get to see their relative before burial or cremation; ambiguous or unresolved grief amongst some bereaved older people or relatives may be a by-product of such funeral practices.

### For older people living in the community

Many of the difficulties regarding staffing and visiting are similar and psychiatric and other day services are closed. The consequent problems of isolation, anxiety, and depression are similar too. Increased suicidal ideation is now an issue. Older people including those with mental health problems are at significant risk of abuse or neglect (Donnelly and O’Loughlin, 2015). Care planning for those with enduring mental health conditions have had to be reviewed to find ways to continue to offer support. As outlined earlier, older people aged over 70 have been “strongly advised” to do what is termed in Ireland, to “cocoon.” Despite the good intentions, there are strong elements of ageism in this policy given that many people over age 70 are fit and well. As Lynch suggests, “we need to remember the rather arbitrary nature of what old age is and how this ‘chronological age’ is a fairly loose benchmark…” (2014: 77). This policy has to be a risk to the mental health of otherwise healthy older people.

### For social workers carrying out their roles

The mechanisms used to offer social work services and support have had to change dramatically during COVID-19. In Ireland, social work has been deemed an essential service, and therefore social workers continue to do their jobs. However, the face-to-face delivery of this service is now much reduced. In its place, online methods of communication beyond telephones are being sought and tested across the country. Maintaining the professional relationships with the users of the social work services remains crucial.

Social workers have changed their work hours and changed work practices to achieve social distancing protocols with colleagues and many now work part-time from home during each week. Some social workers have been temporarily redeployed to undertake new roles or tasks, for example, moving from working with community-based older people to a position as a family liaison worker for nursing homes in a local area. However, in some instances, as mentioned above, redeployment has meant a decrease in frontline social work. The near complete absence of social work services in nursing homes in Ireland has been a problem even before the onset of COVID-19. The use of Internet platforms, such as Zoom, has increased hugely to facilitate meetings, conference calls, and learning opportunities. Such platforms are also being used to give opportunities to social workers to debrief, be supported or get advice from colleagues.

Social workers in residential, hospital, and community settings have sought new creative ways to advocate for and support older people to find ways to overcome social isolation, receive information, and access resources, including increased usage of assistive technology. Social workers have attempted to keep face-to-face contact with older people by offering “walking appointments,” that is, meeting the person at their home, but meeting outside and walking at a physically safe distance together which encourages physical activity, decreases social isolation, and allows the social worker to maintain direct contact.

As referred to above, the issues that COVID-19 has highlighted include dealing with grief and loss in circumstances, where the normal routines have been upset, or in the case of death, the usual rituals cannot be adhered to. Examples of practices by social workers redeployed to nursing homes include facilitating residents to see and talk to family members via social media apps; asking families to purchase tablets for the residents to allow them to maintain regular contact, updating family members on daily basis, preparing family and residents for, and offering support during and after end of life and engaging with funeral undertakers around the particular requirements thrown up by COVID-19, such as having to use cadaver pouches for the deceased.

Social workers in community settings have mobilized volunteers to deliver shopping, meals, medicines, and other necessary items. They are making telephone calls to prompt the taking of medications, to address concerns and maintain contact.

Aside from the work with families mentioned above, social work has continued to support family caregivers in their carer roles. Information giving, linking with agencies and resources, advocacy, and emotional support are key tasks during this pandemic. Many carers are fearful about what should happen if they become unwell and are no longer in a position to provide care for their loved one, so ongoing psychological support is critical. Supporting carers is particularly important insituations where there are pre-existing concerns about safety, for example, in relation to a loved one with dementia. This concern is further heightened when a family member is resistant to intervention. Given that home visiting has largely ceased, social workers are finding new and innovative ways to support carers who are under pressure from their caring role.

At a more global level, the International Federation of Social Workers has worked with national social work organizations and other international agencies to provide opportunities for professional learning and support via webinars and its website. This has reinforced the sense of a global community, that we are all in this together and that people above all else matter in the world.

## Lessons from the experience – Ireland and Europe

As mentioned above, social workers work regularly with individuals and families in crisis situations, so dealing with complex situations is part of daily professional life. This has stood them in good stead in this current crisis, even though it is unprecedented. Social workers are in the frontline in Ireland and across Europe and further afield in supporting older people emotionally and practically to maintain their mental health. This pandemic gives us an opportunity to explore new ways of communicating and offering care to older people.

One of the key issues learned is the need to refocus policy toward the provision of health and social protection services that are comprehensive, integrated, accessible to, and affordable for all. From a European perspective, a greater emphasis must be given to social priorities to balance what have been the dominant economic priorities in European Union policies. This would help to protect the economic and social rights of older people generally and for those with enduring mental illness. The high incidence of and death rates from COVID-19 in nursing homes should be the catalyst for an examination of how we can meaningfully address both societal ageism and the stigma associated with mental health problems.

Responses to the pandemic have shown the similarities between problems in different countries and that we can learn much from each other. In this regard, preparations for a future pandemic must be undertaken so that there is sufficient PPE for professionals to do their jobs safely. It should also be recognized too that staff can have the same anxieties as the people they are attempting to assist. Social workers and other frontline staff must be supported and have opportunities to debrief through good self-care and organizational support strategies.

It is clear that the COVID-19 crisis has thrown up particular challenges in the care of older people with mental illness. It is important to remember that the term crisis refers to change and or opportunity, so change is an important aspect of crisis (Loughran, 2011). We hope therefore that as we transition to a new normal, that we take this unique opportunity to call for the setting up integrated, person-centered long-term care systems in each country (WHO, 2020) which promote human rights prioritize each person’s needs and provide the necessary resources and support to enable all older people with mental illness to age well.
